# Glutaredoxin mediated redox effects of coenzyme Q10 treatment in type 1 and type 2 diabetes patients

**DOI:** 10.1016/j.bbacli.2015.06.001

**Published:** 2015-06-10

**Authors:** Sergio J. Montano, Jacob Grünler, Deepika Nair, Michael Tekle, Aristi P. Fernandes, Xiang Hua, Arne Holmgren, Kerstin Brismar, Johanna S. Ungerstedt

**Affiliations:** a Department of Medical Biochemistry and Biophysics, Karolinska Institutet, Stockholm, Sweden; b Department of Molecular Medicine and Surgery, Karolinska Institutet, Stockholm, Sweden; c Department of Medicine Huddinge, Karolinska Institutet, Stockholm, Sweden; d Department of Environmental Medicine, Karolinska Institutet, Stockholm, Sweden; e Department of Endocrinology, Metabolism and Diabetes, Karolinska University Hospital, Stockholm, Sweden; f Hematology Center, Karolinska University Hospital, Stockholm, Sweden

**Keywords:** CoQ10, coenzyme Q10, DM, diabetes mellitus, Grx1, glutaredoxin 1, GR, glutathione reductase, GSH, glutathione, Kcat, catalytic rate constant, NADP, nicotinamide dinucleotide phosphate, PBMC, peripheral blood mononuclear cell, TAC, total antioxidant capacity, Glutaredoxin, Diabetes mellitus, ROS, Coenzyme Q10, Human

## Abstract

The possible beneficial effects of coenzyme Q10 (CoQ10) supplementation on disease progression and oxidant status in diabetes remains debated. In the present study, patients with type 1 and type 2 diabetes were treated with oral CoQ10, 100 mg twice daily for 12 weeks. We assessed total antioxidant capacity, intra- and extracellular levels of the redox regulating protein glutaredoxin 1 (Grx1), CoQ10, oxidized LDL-cholesterol, lipid profile and HbA1c. We have previously shown that extracellular Grx1 is increased in patients with type 2 diabetes compared to healthy subjects. In the present study, CoQ10 treatment significantly decreased serum Grx1 activity as well as total antioxidant capacity independent of type of diabetes, indicating an improvement to a less oxidized extracellular environment. The effect on serum Grx1 activity was more prominent in patients not on statin treatment. Conversely, intracellular Grx1 activity as well as mRNA levels increased independent of statin treatment. There was a significant improvement in oxidized LDL-cholesterol and lipid profile, with a tendency to improved metabolic control (HbA1c). Additionally, we describe for the first time that CoQ10 is a direct substrate for glutathione, and that Grx1 catalyzes this reaction, thus presenting a novel mechanism for CoQ10 reduction which could explain our findings of an increased intracellular Grx1. In conclusion, 12 weeks CoQ10 treatment significantly improved the extracellular redox balance and lipid profile, indicating that prolonged treatment may have beneficial effects also on clinical outcome in diabetes.

## Highlights

• CoQ10 decreases oxidative stress markers as extracellular Grx1 and oxidized LDL.
• CoQ10 decreases plasma total and LDL-cholesterol in diabetes patients.
• We describe a novel mechanism for in vivo CoQ10 reduction via GSH, catalyzed by Grx1.

## 1 Introduction

Glutaredoxins (Grxs) are redox proteins that catalyze glutathione (GSH) dependent thiol disulfide oxidation reduction reactions in the cytosol or mitochondria [1], [2]. Grx is oxidized by substrate proteins, and subsequently reduced by GSH. Grx also uniquely catalyzes the deglutathionylation of mixed disulfides of proteins with GSH, and by reversible glutathionylation regulates the activity of several proteins, among them apoptosis regulating proteins, e.g. ASK-1 and NFκβ that are found to be dysregulated in diabetes [2]. Patients with diabetes (DM) have an oxidized intracellular environment compared to healthy subjects as hyperglycemia impairs the redox balance and produces ROS [3], [4], which triggers diabetes complications, and DM related morbidity and mortality [5], [6], [7]. We have previously shown that Grx1 plasma activity is increased in type 2 DM compared to healthy subjects, suggesting that extracellular Grx1 activity is a marker of oxidative stress. Additionally, non-diabetic patients had an impaired Grx1 secretion upon excessive glucose stimulation [8]. Thus, serum Grx1 activity may be a prognostic marker in the development of type 2 DM and possibly a marker of DM related oxidative stress which may lead to organ damage. Recent studies have shown that upregulation of Grx1 attenuates revascularization after arterial ischemia [9], implying the importance of low Grx1 levels especially in DM patients who have an increased risk of developing atherosclerosis, and that long term CoQ10 treatment improves morbidity in chronic heart failure.

CoQ10 is a lipid soluble endogenous antioxidant, existing in an oxidized (ubiquinone) and reduced (ubiquinol) form, and a radical intermediate semiquinone. CoQ10 is essential for the mitochondrial electron transport chain that regulates oxidative phosphorylation, and is present in all membranes including the plasma membrane as an antioxidant by inhibiting initiation and propagation of lipid and protein oxidation and regenerating ascorbate and tocopherol [10], [11]. Serum levels of CoQ10 have been reported to be either high or low in type 2 DM compared to healthy subjects [3], [12], [13], where higher levels in DM than in healthy subjects are interpreted to indicate a respons to increased ROS production. Lower levels in DM compared to healthy subjects is reported to be due to deficient production [3], [12], [13]. Preclinical data shows that CoQ10 protects endothelial cells from glucose induced oxidative stress [14], and a number of recent studies in diabetic mice show that CoQ10 protects the mice from developing diabetic nephropaty [15], neuropathy [16], [17], and cardiomyopathy [18]. Additionally CoQ10 protects from β amyloid uptake in a model of Alzheimer's disease [19]. CoQ10 is thus important for neuromuscular function, kidney- and heart function. Several clinical studies have been conducted with CoQ10 supplementation in DM, some showing beneficial effects on clinical parameters [20], [21], [22], while others have not. Recently, a randomized controlled trial of heart failure patients, of whom 10% had diabetes treated with standard therapy or standard therapy with CoQ10 for two years, found that CoQ10 (ubiquinone) treatment reduced the overall cardiovascular-mortality and improved heart function [23]. Similarly, a prospective randomized double blind, placebo controlled trial with CoQ10 and selenium in combination, showed a reduction in long term cardiovascular mortality in a large study of elderly people, of whom 20% had diabetes [24].

In the present study we investigate the possible beneficial effect of dietary supplementation with CoQ10, at 100 mg twice daily, in a population of type 1 and type 2 diabetic patients. The study was not powered by sample size or follow-up time to assess clinical parameters or outcome. The aims were to assess the intracellular and extracellular levels of the oxidative stress parameter Grx1, and to investigate possible interactions between CoQ10 and Grx1.

## 2 Material and methods

TrxR1 enzyme, hGrx1 and fluorescent Grx1 activity kit were from IMCO (IMCO Corporation Ltd; www.imcocorp.se), CoQ10 (ubiquinone) was a gift from Pharma Nord (Vejle, Denmark), NADPH and all other reagents were purchased from Sigma-Aldrich (St. Louis, Missouri, USA).

### 2.1 Patients

22 patients, diagnosed with type 1 or type 2 DM according to WHO guidelines [25], were enrolled in the study at the Department of Endocrinology, Metabolism and Diabetes, Karolinska University Hospital, Stockholm, Sweden, after giving informed written and oral consent. The study was approved by the regional Ethics Committee in Stockholm, and was carried out in accordance with the Declaration of Helsinki.

### 2.2 CoQ10 administration and sample collection

After overnight fasting, venous blood was drawn from the antecubital vein, at study enrollment and after 12 weeks of oral CoQ10 (ubiquinone) treatment. All patients received 100 mg CoQ10 supplementation twice daily for 12 weeks. Heparin tubes were collected and PBMC separated using a Ficoll gradient centrifugation, and PBMC quickly frozen at − 20 °C until analysis. Heparin tubes were centrifuged 20 min at 2500 ×*g*, where after plasma was collected quickly frozen and kept at − 80 °C until further analysis.

### 2.3 Routine laboratory investigations

The lipid profile and glycemic status were determined according to standard procedures at the Karolinska University Hospital Clinical Laboratory (www.diagnosticsample.com), using the automated DXC Beckman-Coulter instrument (www.beckmancoulter.com). HbA1c was determined using the MonoS method, Unimate (Roche Diagnostics, Basel, Switzerland). To convert HbA1c MonoS into HbA1c NGSP, the formula NGSP = 0.92 ∗ MonoS + 1.33 is used.

### 2.4 Determination of oxidized LDL

The plasma concentration of oxidized LDL was determined by commercially available sandwich ELISA according to the manufacturer's description (Mercodia AB, Uppsala, Sweden).

### 2.5 CoQ10 detection

CoQ10 was extracted from the samples according to Bentinger et al. [26] with some modifications. 200 μL serum and 300 μL water were mixed, 5 mL methanol was added and the sample was vortex-mixed for 1 min, 1.2 mL petroleum-ether was added and shaken by hand for about 15 s. Finally, phase separation was obtained by adding 2 mL methanol and 1.8 mL petroleum-ether and where after the sample was shaken again for 15 s, where after the samples were centrifuged, and the upper phase was transferred to a new tube and evaporated under nitrogen.

Samples were dissolved in chloroform:methanol 2:1 and a portion injected for reversed phase separation on HPLC using a C18 column (150 × 3.9 mm, 5 μm particle size, Waters Millipore, MA) equipped with a Supelco C8 pre-column (50 × 3.9 mm, 5 μm, Waters Millipore). A linear gradient was used from methanol:water 9:1 to methanol:isopropanol:hexane 2:1:1 for 25 min with UV-detection at 210 and 275 nm. Peaks were identified with corresponding standards.

### 2.6 Grx1 activity assay in serum and PBMC

Grx1 activity in serum or PBMC was determined with a commercially available Fluorescent Grx1 activity assay kit (IMCO Corporation Ltd; www.imcocorp.se) developed in our group [8]. In brief, 20 μL of serum or 40 μL PBMC lysate was mixed thoroughly with 70 μL of 100 mM potassium phosphate buffer, pH 7.5, containing 1 mM EDTA, 0.5 mM GSH, 0.25 mM NADPH, 50 nM baker yeast and glutathione reductase (GR) in a 96-well plate. Then 10 μL of fluorescent albumin (BSA-S-SG-E) was then added to each well to initiate the reaction. A reaction solution containing plasma/PBMC lysate but not GSH, NADPH, or GR was used as background. The emission at 540 nm was recorded after 520 nm excitation using a VICTOR^3^ Multilabel Plate Reader (PerkinElmer, USA). Grx1 activity was determined by measuring the initial reaction velocity from a linear fluorescence increase. Varied concentrations of recombinant hGrx1 (IMCO Corporation Ltd AB, Stockholm, Sweden) were used to obtain a standard curve, based on which the corresponding Grx activity was calculated. We approximate that essentially all serum Grx activity consists of Grx1 activity, as previously described [27].

### 2.7 Glutathione-dependent CoQ10 reduction catalyzed by Grx1

In vitro experiments of ubiquinone were carried out by adding up to 75 μM CoQ10 to a reaction mixture containing 10 mM GSH, 10 nM GR and 0.2 mM NADPH recording the NADPH oxidation spectrophotometrically at 340 nm. To study the catalyzed reaction, 20 μM Grx1 was added to the reaction mixture.

### 2.8 Thioredoxin reductase (TrxR1) catalyzed GSH-dependent CoQ10 reduction

To compare the previously described CoQ10 reduction catalyzed by TrxR [28] to that of GSH and Grx1, and to determine the reaction rate under our experimental setup, up to 100 μM CoQ10 was added to a reaction mixture containing a final concentration of 100 nM TrxR1 and 0.2 mM NADPH, where after NADPH oxidation was recorded with a spectrophotometer at 340 nm for 30 min.

### 2.9 Total antioxidant capacity (TAC) assay

TAC was measured with a spectrophotometer in 96-well plates, using the Antioxidant Assay Kit (Sigma) as per the manufacturer's instructions. After 5 min incubation, reaction was stopped and the endpoint absorbance at 405 nm was determined using a microplate reader (SpectraMax 340PC, 384 — Molecular Devices). All samples were performed in duplicate. A reference curve based on the soluble antioxidant Trolox was used, and TAC was expressed in Trolox concentration (mM).

### 2.10 RNA extraction and quantitative PCR

Total RNA was isolated and cDNA was reversely transcribed using Omniscript Reverse Transcription Kit protocol (Qiagen, Hilden, Germany) according to the manufacturer's instructions, followed by quantitative PCR (qPCR) for Grx1 (primer sequences forward: TGCAAAATCCAGCCTGGGAAG, reverse: TTGAATCTCGTTAGTGTGGTTGGT. β-Actin was used as a housekeeping gene. qPCR was carried out using SYBR Green qPCR Super Mix (Invitrogen, Carlsbad, CA, USA) in Bio-Rad iCycler (Bio-Rad, Hercules, CA). The expression ratio was calculated using the ^ΔΔ^CT method. cDNA, SYBR Green PCR Master Mix (Invitrogen, Carlsbad, CA, USA), and forward and reverse primers at 300 nM concentration were added to each well. Specificity of the PCR reaction was validated by melting curve analysis. All qPCRs were performed in duplicates.

### 2.11 Statistical analyses

The Wilcoxon matched pair test was used to compare 0 weeks and 12 weeks, assuming non-Gaussian distribution of samples. The difference between groups was compared with Mann–Whitey U test. Differences were considered to be significant if p < 0.05.

## 3 Results

### 3.1 Patient characteristics and effect of CoQ10 on clinical parameters

Of the 22 patients enrolled in the study, 13 had type 1 DM and 9 had type 2 DM. Patient characteristics are described in Table 1, reference levels given in the table legend. Statin use was more frequent in the type 2 DM group (67% of type 2 DM patients were on statin treatment compared to 31% of type 1 DM patients), and the baseline lipid profile was significantly dysregulated in the type 2 DM group. Baseline serum CoQ10 levels were similar between the groups, and higher than found in healthy individuals [8]. Serum CoQ10 levels increased significantly upon treatment, with no difference between statin treated or non-statin treated patients (Table 2).

Subgroup analysis of the 11 statin treated and 11 non-statin treated patients showed that even though there were no significant differences at baseline lipid status between statin treated and non-statin treated patients, p-LDL, oxidized LDL, and total p-cholesterol decreased significantly only in the non-statin treated group. However, the CoQ10/cholesterol ratio decreased significantly in both subgroups, and HbA1c decreased significantly only in the statin treated group, where actually all patients individually decreased their HbA1c (Table 2).

### 3.2 Serum Grx1 activity and total antioxidant capacity

**Fig. 1:**
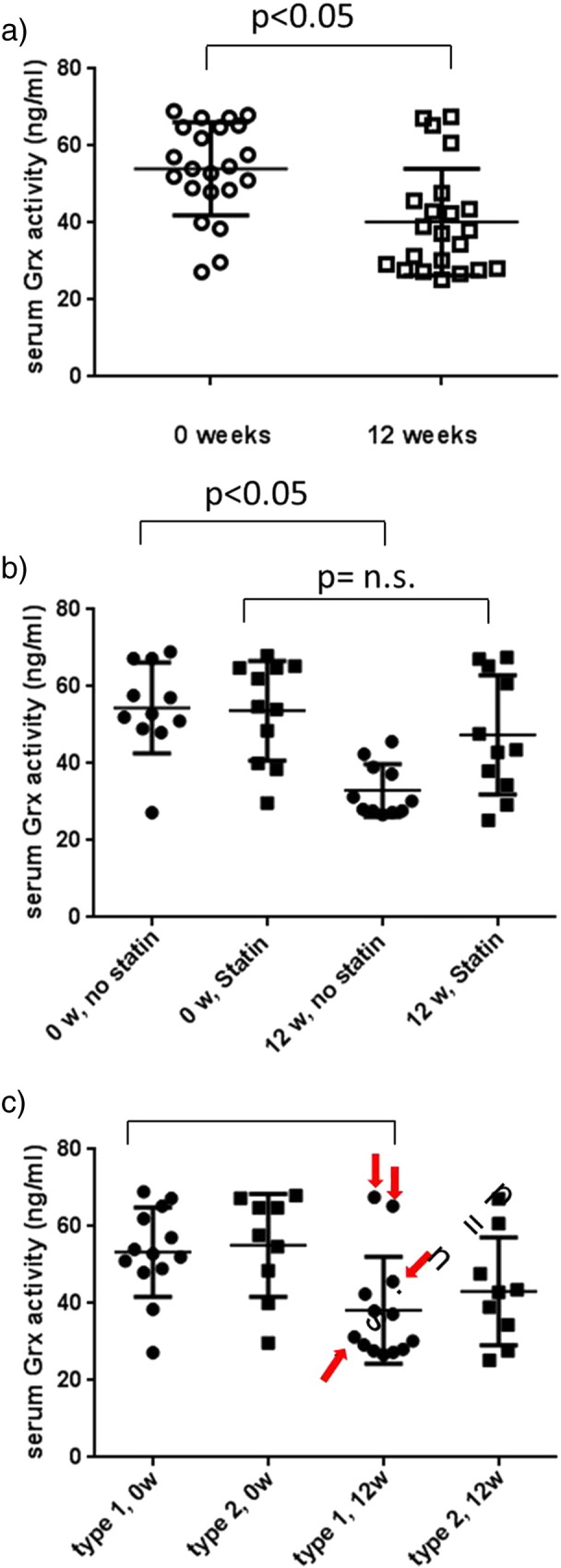
Serum Grx1 activity in 22 diabetes patients before and after 12 weeks of CoQ10 treatment. Mean and SD are shown. Serum Grx1 activity significantly decreased upon CoQ10 treatment in the whole patient cohort (a). The decrease was significant in non-statin treated patients (p < 0.05) but not significant in the statin treated cohort (b). The decrease in Grx1 activity was similar regardless of the type of DM (c), however two of the four type 1 DM patients on statin treatment had no decrease in Grx in response to CoQ10 (statin treated type 1 DM patients marked with red arrow).

Serum Grx1 activity decreased significantly after 12 weeks of CoQ10 treatment, from a median of 54 ng/mL (range 27–69) to 38 ng/mL (range 25–68), p < 0.05 (Fig. 1a and Table 2). Patients were further subdivided according to the statin treatment or not (Fig. 1b), and type of DM (Fig. 1c), to assess in which patient subgroup the decrease in Grx1 activity was most prominent. The results clearly show a marked reduction in extracellular Grx1 activity in non-statin treated patients, however not in statin treated patients (Table 2, Fig. 1b). Since only 4 of 13 type 1 DM patients were on statin treatment compared to 7 of 9 type 2 DM patients, it is difficult to assess the importance of DM type on Grx1 levels, however it is worth noting that two of the four DM type 1 patients on statin treatment did not decrease their Grx1 levels at all (Fig. 1c).

**Fig. 2:**
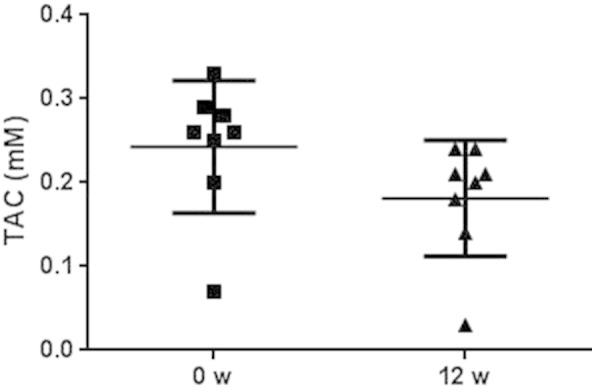
Serum total antioxidant capacity (TAC) was measured in eight representative patients. Of these, four had type 1 DM and four had type 2 DM, and four of the eight patients were on statin treatment. As seen for serum Grx1 activity, there was a significant decrease in TAC after 12 weeks of CoQ10 treatment (p < 0.05).

To confirm the above result, total antioxidant capacity (TAC) was measured if remaining sample amount allowed (n = 8 of which four type 1 DM, four type 2 DM; four of these patients were on statin treatment). Similar to the decrease in plasma Grx1 activity and oxidized LDL cholesterol, there was a significant decrease in plasma TAC (p < 0.05), Fig. 2.

### 3.3 Intracellular Grx1 activity and mRNA levels

**Fig. 3:**
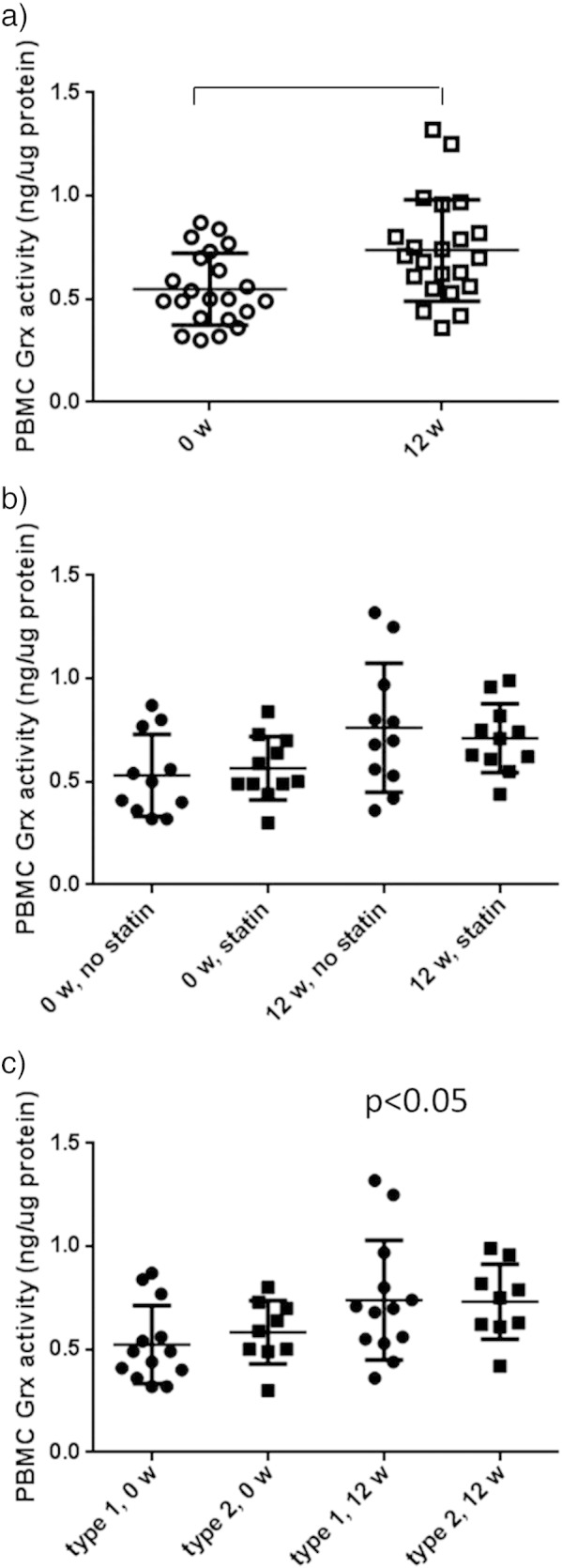
Intracellular PBMC Grx activity before and after 12 weeks of CoQ10 treatment. A significant increase in intracellular Grx activity of the 22 patients was seen. When analyzing the subgroups depending on statin treatment (b) or diabetes subtype (c), there was no difference in the PBMC response depending on statin treatment or diabetes type.

**Fig. 4:**
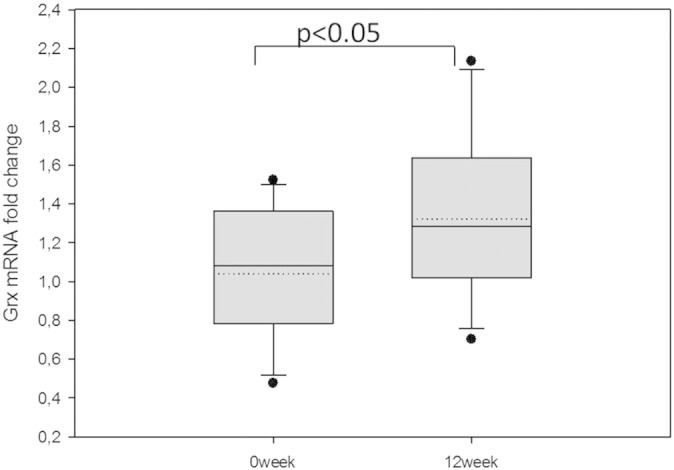
PBMC mRNA expression of Grx1 increased significantly (p < 0.05) upon 12 weeks of CoQ10 treatment, n = 22.

When analyzing intracellular PBMC Grx1 activity, we found a significant increase after 12 weeks of CoQ10 treatments (Table 2, Fig. 3a). The increase was similar in statin treated patients as well as in patients not on statin treatment (Fig. 3b), and similar in type 1 and type 2 DM patient groups (Fig. 3c). To confirm the results, we analyzed Grx1 mRNA expression in the same PBMC samples, and in line with the increase in Grx1 activity, there was also an increase in Grx1 mRNA expression (Fig. 4), after 12 weeks of CoQ10 treatment.

### 3.4 Evidence for a GSH-dependent CoQ10 reduction, catalyzed by Grx1

**Fig. 5:**
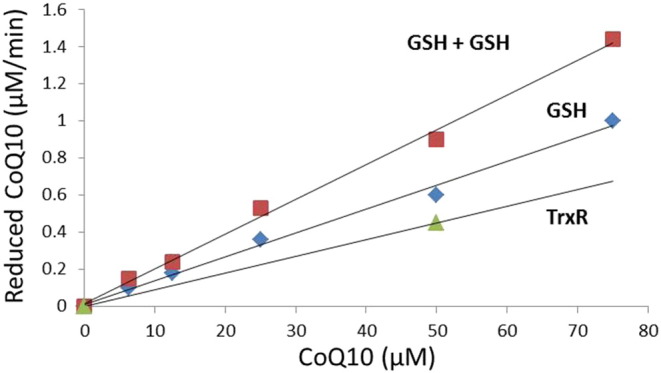
Amount of CoQ10 reduced, with either 10 mM GSH, 0.2 mM NADPH and varying CoQ10 concentration, with and without 20 μM Grx1, or 0.1 μM TrxR, 0.2 mM NADPH with 50 μM CoQ10. We assume that 1 mol oxidized NADP + equals 1 mol reduced CoQ10. GSH is a better reductant than TrxR, and the reaction is catalyzed by the addition of Grx1.

The results from serum and intracellular Grx1 activity measurements suggested that there may be a link between CoQ10 and Grx1, either by CoQ10 being directly reduced by Grx1 or part of the reduction process. We tested our hypothesis by incubating different concentrations of CoQ10 with GSH (10 mM) in the presence of GR (10 nM), NADPH (0.2 mM), with or without 20 μM Grx1. We found that GSH reduces CoQ10 quite comparable to TrxR, and that the reduction of CoQ10 is catalyzed by the addition of Grx1 (Fig. 5). We assume that 1 mol NADP^+^ produced equals 1 mol CoQ10 reduced. Because of the turbidity caused when adding more than 100 μM CoQ10, we were not able to reach saturation of the reaction, thus a Lineweaver–Burk plot could not be created and Km could not be determined. To compare the catalytic efficiency of this novel mechanism, with the previously described TrxR mediated CoQ10 reduction [28], we repeated the experiments of Xia et al. [28]. In our experimental setup, we used 0.2 mM NADPH, 100 nM final concentration TrxR, and 50 μM CoQ10. We assume that 1 mol NADP^+^ produced equals 1 mol CoQ10 reduced, as above. The result is 0.45 μM reduced CoQ10 per minute. Comparing the catalytic activity, this equals a Kcat of 4.5 per minute per TrxR molecule, whereas the Kcat of Grx1 is 0.05 per minute per Grx1 molecule. This low Kcat of Grx1 is however misleading, as it to a large extent may be explained by the high background reducing activity of GSH. When comparing the amount of reduced CoQ10 depending on CoQ10 concentration, the GSH/Grx system is actually a slightly better reductant (Fig. 5).

## 4 Discussion

In the current study, we found that 12 weeks of oral CoQ10 treatment decreased extracellular Grx1 activity and total antioxidant capacity as well as fp-LDL, oxidized LDL cholesterol, and total cholesterol, indicating an improvement towards a less oxidized extracellular environment. The findings are in line with our previous proposal that extracellular Grx1 is a predictor of impaired extracellular redox balance and impaired coping with hyperglycemia induced oxidative stress, thus a risk marker for DM onset and progression [8], and the finding that increased Grx1 attenuates post-ischemia revascularization in a mouse model [9]. When analyzing statin treated and non-statin treated subgroups in the CoQ10 patients, the positive changes in redox parameters and lipid status were only significant in the non-statin treated population. In our study, baseline CoQ10 levels were in the same range as previously described [12], [29]. CoQ10 treatment increased serum CoQ10 levels significantly in all patients. In fact, the increase was slightly higher than expected for 12 weeks CoQ10 treatment, even if other studies also have described a threefold increase in CoQ10 levels [21]. Possibly, the high levels were achieved due to the quite high levels of circulating p-LDL in the patients, binding to CoQ10 and thus keeping high levels of CoQ10 in the circulation. Serum CoQ10 levels increased regardless of statin treatment and type of DM, thus, there must be other factors determining that the positive antioxidant response only occurred in non-statin treated patients.

In the current study we measured serum CoQ10 levels and not intracellular levels, and as it is well known that serum CoQ10 levels do not always reflect intracellular CoQ10 levels [30], we speculate that CoQ10 cellular uptake may be somehow perturbed in statin treated patients rendering lower intracellular levels, which would explain the lack of positive antioxidant effects in the statin treated patients. In support of this, it has been shown that although shorter time statin treatment does not alter the intracellular production of CoQ10 [31], [32], long time statin treatment does decrease CoQ10 serum levels [33]. In fact, statin treatment has been reported to be a moderate risk factor for developing type 2 diabetes [34].

Recently, two large clinical studies have been performed on the effects of CoQ10 supplementation. The first was a population based prospective, randomized double blind placebo controlled study of CoQ10 and selenium supplementation, showing a decreased mortality at 5 year follow-up [24], and the second was a randomized controlled study of CoQ10 in chronic heart failure, where CoQ10 treatment improved symptoms and reduced cardiovascular events [23]. Although the majority of the patients did not have diabetes, a significant proportion of patients were on statin treatment. These studies show that high serum CoQ10 levels (similar levels that we report in the current study) are needed for a protective effect on cardiovascular events and mortality. This suggests that even if basal CoQ10 levels are increased by 50–75% in diabetes compared to healthy controls, the endogenous levels are too low to reduce oxidative stress. In our study, oxidized LDL, fp-LDL and cholesterol levels were significantly reduced upon CoQ10 treatment, and HbA1c levels tended to improve, thus indicating a possible beneficial effect also in clinical parameters, despite the small patient size and relatively short treatment period.

Additionally, we describe for the first time that Grx catalyzes the reduction of CoQ10 by GSH. As TrxR has previously been demonstrated to reduce CoQ10 [28], we assessed this TrxR catalyzed CoQ10 reduction in our experimental system and found Kcat similar to the Grx1 catalyzed GSH reduction of CoQ10. These results show a new mechanism for in vivo CoQ10 reduction. Additionally, at physiological concentrations (GSH 10 mM, Grx1 20 μM, and TrxR 0.1 μM) it may well be that GSH and Grx1 are more important for reducing CoQ10 than TrxR.

In the present study, we found that as the extracellular Grx1 decreased by CoQ10 treatment, intracellular Grx1 levels as well as Grx1 mRNA increased. The reason behind the intracellular Grx increase is unclear and warrants further investigation, where the main intracellular antioxidant glutathione (GSH), present at mM concentrations in the cell and known to be decreased in type 2 diabetes compared to healthy [35], should be assessed as well as the balance between its oxidized (GSSG) and reduced (GSH) state, changes of which may affect intracellular as well as extracellular Grx levels.

In conclusion, we have described that markers of oxidative stress, including extracellular Grx1 and oxidized LDL, are reduced upon treatment of DM patients with CoQ10. Moreover, we have described a novel mechanism for in vivo CoQ10 reduction via GSH, catalyzed by Grx1. The positive effects of CoQ10 treatment on redox parameters as well as on the lipid profile and metabolic control may have clinical implications in DM.

## Conflict of interest

The authors declare no conflict of interest.

## Transparency Documents

Transparency documents.

## Figures and Tables

**Table 1:** Patient characteristics at baseline. The type 1 and type 2 DM patients had similar baseline characteristics, regarding age, gender, clinical parameters and biochemical parameters, except for diabetes duration, lipid profile, and BMI. There was a significantly higher use of statin treatment and in type 2 diabetes patients. No significant differences were observed in baseline CoQ10 levels, intracellular or extracellular Grx levels between type 1 and type 2 DM patients. Reference levels for healthy population are HbA1c% < 5.1%, fp-TG 0.45–2.6 mmol/L, fp-HDL 1.0–2.7 mmol/L, fp-LDL cholesterol 2.0–5.3 mmol/L, and total cholesterol < 5 mmol/L. For Grx, we previously reported serum Grx levels of 15.2 ng/mL in healthy individuals (8). There is no established reference range for oxidized LDL or PBMC Grx.

	Type 1 diabetes	Type 2 diabetes	p-Value
Age (median, range)	57 (35–71)	63 (52–70)	ns
Gender (m/f)	6/7	6/3	
Statin (yes/no)	4/9	7/2	p < 0.05
Years of disease (median, range)	31 (8–53)	8(3–38)	p < 0.05
HbA1c % MonoS (median, range)	6.7 (4.5–9.0)	6.4 (5.0–8.4)	ns
BMI (median, range)	23.4 (21.6–35.6)	28.6 (23.7–41)	p = 0.004
Blood pressure (median, range)	128/80 (110–144/65–93)	125/80 (120–140/60–98)	ns
fp-TG, mmol/L (median, range)	0.64 (0.4–2.5)	1.3 (0.87–2.0)	p < 0.05
fp-HDL, mmol/L (median, range)	1.8 (0.8–2.5)	1.0 (0.7–2.0)	p < 0.01
fp-LDL cholesterol, mmol/L (median, range)	2.3 (1.9–4.3)	2.6 (1.6–2.9)	ns
Serum CoQ10, nmol/L(median, range)	1.70 (1.1–3.3)	1.75 (1.0–3.4)	ns
Total cholesterol, mmol/L(median, range)	4.9 (3.6–6.2)	4.4 (3.1–5.4)	ns
CoQ10/cholesterol (median, range)	0.35 (0.20–0.92)	0.48 (0.29–0.77)	ns
Oxidized LDL cholesterol (nM)	37.3 (24.5–63.3)	48.6 (22.2–75.5)	ns
Serum Grx (ng/mL)	52.9 (27.2–69)	57.7 (29.8–68)	ns
PBMC Grx (ng/μg)	0.5 (0.32–0.87)	0.6 (0.30–0.80)	ns

**Table 2:** Effects of 12 weeks of CoQ10 treatment on CoQ10, Grx, HbA1c and lipids in 22 patients with DM, analyzed in non-statin treated (n = 11) and statin treated (n = 11) separately, and all together. Median, range are given and significance at the level of p < 0.05 (*), or < 0.01 (**). Upon CoQ10 treatment, there was a significant reduction in serum Grx, fp-LDL-cholesterol, total p-cholesterol, as well as oxidized LDL-cholesterol, with more pronounced changes in the non-statin treated group. There was a tendency to reduced HbA1c levels in the whole patient population, and interestingly a significant increase in the statin treated group where in fact all 11 patients individually lowered HbA1c. Intracellular Grx increased significantly, and serum CoQ10 increased.

	Non-statin treated		Statin treated		All patients	
0 w	12 w	0 w	12 w	0 w	12 w
S-CoQ10 (mmol/L)	1.8 (1.1–3.4)	5.1 (4.1–7.3)⁎⁎	1.5 (1.0–2.8)	4.0 (3.4–9.0)⁎⁎	1.7(1.0–3.4)	5.0(3.4–9.0)⁎⁎
S-Grx (ng/mL)	52.9 (27–69)	30.2 (26–45)⁎⁎	54.7 (29–68)	43.6 (25–67)	54 (27–69)	38 (25–67)⁎
Grx PBMC (ng/μg prot)	0.5 (0.32–0.87)	0.7 (0.44–0.99)	0.5 (0.3–0.84)	0.7 (0.44–0.99)	0.5 (0.3–0.9)	0.7 (0.4–1.3)⁎
HbA1c^a^ (%)	6.7 (5.4–9.0)	6.5 (5.5–8.3)	6.0 (4.5–8.4)	5.7 (4.3–7.6)⁎	6.5 (4.5–9)	5.9 (4.3–8.3)
p-HDL (mmol/L)	1.4 (0.9–2.5)	1.4 (1.9–2.4)	1.0 (0.8–2.3)	1.0 (0.8–1.8	1.2 (0.7–2.5)	1.3 (0.7–2.4)
p-LDL (mmol/L)	2.5 (2.0–4.3)	2.2 (1.7–3.2)⁎	2.3 (1.6–3.9)	2.1 (1.6–3.4)	2.4 (1.6–4.3)	2.2 (1.4–3.4)⁎
p-Triglycerides (mmol/L)	0.7 (0.4–2.5)	0.7 (0.4–1.5)	1.3 (0.5–2.0)	1.3 (0.6–2.0)	1.0 (0.4–2.5)	1.0 (0.4–2)
Ox. LDL	38.2 (24–63)	34.2 (22–47)⁎⁎	38.3 (22.-75)	36.7 (29–49)⁎	38.2 (22–75)	35.9 (22–49)⁎⁎
p-Total cholesterol	4.9 (3.6–6.2)	4.3 (3.9–5.2)⁎⁎	4.4 (2.9–5.4)	3.9 (2.7–5.8)	4.6 (3.1–6.2)	4.0 (2.5–5.8)⁎
CoQ10/cholesterol	0.35 (0.22–0.92)	1.15 (0.84–1.8)⁎⁎	0.42(0.29–0.63)	1.25 (0.76–2.6)⁎⁎	0.45 (0.22–0.92)	1.24 (0.76–2.6)⁎

Reference levels for healthy population are HbA1c% < 5.1%, fp-TG 0.45–2.6 mmol/L, fp-HDL 1.0–2.7 mmol/L, fp-LDL 2.0–5.3 mmol/L, and total p-cholesterol < 5 mmol/L. For Grx, we previously reported serum Grx levels of 15.2 ng/mL in healthy individuals (8). There is no established reference range for oxidized LDL or PBMC Grx.

a HbA1c measured with the MonoS method.

⁎ p < 0.05 for 12 w vs. 0 w.

⁎⁎ p < 0.01 for 12 w vs. 0 w.
